# Six-month cost-effectiveness of adding motivational interviewing or a stratified vocational advice intervention to usual case management for workers with musculoskeletal disorders: the MI-NAV economic evaluation

**DOI:** 10.1186/s12995-023-00394-2

**Published:** 2023-11-14

**Authors:** Alexander Tingulstad, Esther T. Maas, Tarjei Rysstad, Britt Elin Øiestad, Fiona Aanesen, Are Hugo Pripp, Maurits W. Van Tulder, Margreth Grotle

**Affiliations:** 1 Department of Rehabilitation and Health Technology, Centre for Intelligent Musculoskeletal Health, Oslo Metropolitan University, St.Olavs Plass, P.O. Box 4, Oslo, 0130 Norway; 2 Department of Health Sciences, Faculty of Science, Vrije Universiteit Amsterdam, and the Amsterdam Movement Sciences Research Institute, de Boelelaan 1085, Amsterdam, 1081 HV The Netherlands; 3 National Institute of Occupational Health, Majorstuen, P.O. Box 5330, Oslo, 0304 Norway; 4 Faculty Behavioural and Movement Sciences, Vrije Universiteit Amsterdam, Van Der Boechorststraat 7, Amsterdam, 1081 BT The Netherlands; 5 Department of Research, Innovation and Education, Division of Clinical Neuroscience, Research and Communication Unit for Musculoskeletal Health (FORMI), Oslo University Hospital, Ullevål, Building 37B, P.O. Box 4956, Oslo, Nydalen 0424 Norway

**Keywords:** Vocational rehabilitation, Economic evaluation, Return to work, Sick leave, Randomized controlled trial

## Abstract

**Objectives:**

This study evaluates the six-month cost-effectiveness and cost-benefits of motivational interviewing (MI) or a stratified vocational advice intervention (SVAI) added to usual case management (UC) for workers on sick leave due to musculoskeletal disorders.

**Methods:**

This study was conducted alongside a three-arm RCT including 514 employed workers on sick leave for at least 50% for ≥ 7 weeks. All participants received UC. The UC + MI group received two MI sessions, and the UC + SVAI group received 1–4 SVAI sessions. Sickness absence days, quality-adjusted life-years (QALYs), and societal costs were measured between baseline and six months.

**Results:**

Adding MI to UC, resulted in incremental cost-reduction of -2580EUR (95%CI -5687;612), and a reduction in QALYs of -0.001 (95%CI -0.02;0.01). Secondly, adding MI to UC resulted in an incremental cost-reduction of -538EUR (95%CI -1358;352), and reduction of 5.08 (95%CI -3.3;13.5) sickness-absence days. Financial return estimates were positive, but not statistically significant. Adding SVAI to UC, resulted in an incremental cost-reduction of -2899 EUR (95% CI -5840;18), and a reduction in QALYs of 0.002 (95% CI -0.02;0.01). Secondly, adding SVAI to UC resulted in an statistically significant incremental cost-reduction of -695 EUR (95% CI -1459;-3), and a reduction of 7.9 (95% CI -0.04;15.9) sickness absence days. Financial return estimates were positive and statistically significant. The probabilities of cost-effectiveness for QALYs were high for adding MI or SVAI (ceiling ratio 0.90).

**Conclusions:**

In comparison to UC only, adding MI to UC tends to be cost-effective. Adding SVAI to UC is cost-effective for workers on sick leave due to musculoskeletal disorders.

**Trial registration:**

ClinicalTrials.gov (identifier: NCT03871712).

**Supplementary Information:**

The online version contains supplementary material available at 10.1186/s12995-023-00394-2.

## Introduction

Musculoskeletal disorders (MSDs) are the main reason for disability worldwide [1]. In Europe, MSDs are also the most frequent cause of reduced work productivity and sickness absence [2, 3]. Sickness absence is associated with significant costs for individuals and society [2, 4]. Furthermore, staying at work or returning to work after sickness absence is essential for a persons’ identity, social role and status in society [5]. To address sickness absence and the large economic burden related to this, effective and cost-effective interventions targeting barriers to return to work (RTW) are needed [6].

Finding effective RTW interventions is difficult due to the complexity of long-term sick leave. Interactions between individual, workplace, healthcare, compensation system and societal factors may hamper RTW [7]. Two potential interventions that have shown to be promising to reduce sick leave duration are Motivational Interviewing (MI) and Stratified Vocational Advice Intervention (SVAI). MI is a person-centred counselling style aimed at increasing motivation for change [8]. The SVAI intervention was based on the principles of case management to help participant overcome obstacles to RTW [9].

A recent trial (MI-NAV) [10, 11] studied the additional effectiveness of either MI or SVAI to usual case management (UC) to reduce sickness absence days over a six-month period. This study focused on workers with MSDs who had been on sick leave for more than seven consecutive weeks. Adding MI or SVAI to UC reduced sickness absence by an average of seven workdays over six months. Although seven days seems a relevant difference, it was not statistically significant and the wide confidence intervals (CIs) indicated imprecise estimates [11].

Considering the limited resources for RTW interventions, stakeholders are interested in the effectiveness as well as if it is worth their money before implementation [12]. Economic evaluations provide such information by estimating the difference in effects and costs between two or more interventions, and relate those to each other [13].

Therefore, this study aimed to evaluate the six-month cost-effectiveness, cost-utility, and cost–benefit of adding MI to UC, and adding SVAI to UC for workers who have been on sick leave for at least seven weeks due to MSDs.

## Methods

The methods have been previously reported in the study protocol [10], and the publication of clinical effectiveness [11]. We followed the International Society for Pharmacoeconomics and Outcomes Research (ISPOR) Cost-Effectiveness Analysis Randomised Clinical Trial taskforce recommendations [14].

### Study design

An economic evaluation was conducted alongside a three-arm, pragmatic RCT [10]. The trial included participants between April 2019 and October 2020. As well as the economic evaluation, the trial had a follow-up at six months. The trial was conducted in cooperation with the Norwegian Labour and Welfare Administration (NAV). The project was approved by the Norwegian Centre for Research Data (project nm. 861249), and the trial was conducted according to the Helsinki declaration and the General Data Protection Regulation.

### Participants, randomisation, and stratification

Eligible participants were workers aged 18–67 years, employed either full or part-time, and on sick leave due to MSDs for at least 50% of their contracted work hours for more than seven consecutive weeks. All participants were diagnosed with MSDs listed in the 2^nd^ edition of the International Classification of Primary Care (ICPC-2) [15]. Excluded were those with serious somatic or mental health disorders affecting their work ability and in need of specialised treatment (e.g., cancer, psychotic disorders), pregnant women, unemployed, freelancers and self-employed workers, and those lacking sufficient proficiency in either Norwegian or English to answer questionnaires or communicate by telephone.

Candidates who agreed to participate received an electronic link to written information about the trial, an electronic informed consent form and a baseline questionnaire.

The Örebro Musculoskeletal Pain Screening Questionnaire Short Form (ÖMPSQ-SF) [16], and the Keele STarT MSK Tool [17, 18] were used to stratify the participants into two risk groups of long-term sick leave. Participants with ≥ 9 on the Keele STarT MSK Tool and ≥ 60 on the ÖMPSQ-SF were stratified to a ‘high-risk group’, all others were stratified to a ‘medium/low-risk group’. After stratification, participants were randomised using a 1:1:1 ratio. Allocation was concealed for the recruitment staff. A blinded statistician prepared a computer-generated allocation sequence for each risk-group, only available for the person in charge of group allocation.

### Interventions

A detailed description of the rationale, development and content of the intervention can be found elsewhere [10, 11]. A fidelity assessment of the MI intervention [19], and a process evaluation of the SVAI have been published previously [20]. All participants received UC for sick leave, consistent with Norway’ standard practice, which provides full wage replacement benefits for up to 12 months. The usual case management has the following timeline: within the first 4 weeks of sick leave, an RTW plan is made by the employer and employee; within 7 weeks, a dialogue meeting between the employee, employer, and other relevant stakeholders such as general practitioner (GP), is arranged by the employer. Within week 26 of the sick leave period, NAV arranges a second dialogue meeting between the employee, the employer and in some cases the GP who issued the sick leave.

In addition to UC, participants randomised to the UC + MI arm were offered two face-to-face sessions of MI from a NAV caseworker; the first as soon as possible after random allocation, and the second two weeks later. The NAV caseworkers were educated in MI [10].

The participants in the UC + SVAI arm were offered UC and vocational advice and case management from physiotherapists. In the UC + SVAI group, those stratified to the low/medium-risk group were offered 1–2 telephone sessions, while participants in the high-risk group were offered 3–4 sessions. The first session was conducted as soon as possible after inclusion, and the intervention ended when the participant reached six months of consecutive sick leave or had RTW for four consecutive weeks. Eight physiotherapists were trained over a five-day course to provide SVAI.

### Effect measures

The primary effect measure was the number of sickness absence days over a six-month period, defined as lost workdays. To accurately represent time away from work, we accounted the participants’ contracted work hours and amount of sick leave. This was then summed up and converted to lost workdays, assuming a five-day working week. Data was obtained from national registries, including information on sick leave benefits, sick leave certificates, disability pensions, and contracted work hours. In Norway, people may work alongside part-time disability pensions, so any increase in disability pensions from baseline was counted as sick leave.

The secondary effect measure was health-related quality of life expressed in terms of quality adjusted life years (QALYs). First, the participants’ health states were measured by the EuroQol-5 Dimensions-5 Levels (EQ-5D-5L) [21]. Then, the UK tariff was used to convert these health states into utility scores, anchored at 0 “death” and 1 “perfect health”, with negative values representing health states worse than death. We used the UK tariff, as a Norwegian tariff is not available. QALYs were calculated using the “area under the curve” approach. The willingness-to-pay threshold for this outcome was based on the Norwegian governmental report No. 34 to the parliament with a value of NOK 275,000 (Euro (€) 27,500/USD 35,628) per QALY [22].

### Cost measures

Since this study adopted a societal perspective, we included both direct and indirect costs. Direct costs included costs of the intervention, primary healthcare use (e.g., general practitioner, physiotherapist, manual therapist, or other therapists), and secondary healthcare use (e.g., hospitalisation or rehabilitation). To calculate intervention costs, we employed a micro-costing approach and included training and mentoring costs. Intervention costs were provided per hour by NAV. Information on other health care use and costs was retrieved from national registers: The Norwegian Health Economics Administration and the Norwegian Patient Registry. Indirect costs consisted of work absenteeism and productivity losses due to paid and unpaid work. We obtained absenteeism data from national registries and valued it using estimates from official statistics on average income stratified by gender. Productivity losses due to unpaid work were measured using the Institute for Medical Technology Assessment Productivity Cost Questionnaire (iPCQ) [23]. The iPCQ has been translated and culturally adapted to Norwegian and found to have good measurement properties when used among patients with long-term MSDs [24]. These costs were valued using a recommended Norwegian shadow price (€150). All costs were converted to 2021 Euros, the last year of data collection, using exchange rates from the European Central Bank. Since the follow-up period of the intervention was less than one year, there was no need to discount the costs and effects.

### Statistical analyses

Analyses were performed in accordance with the published statistical analysis plan [10]. All analyses were performed according to the intention to treat principle. Unless stated otherwise, data were analysed using Stata (version 16, Stata Corp, College station, TX).

#### Missing data

We anticipated few missing values for the primary outcome and the work-related secondary outcomes, as information was obtained from the Norwegian national social security system registry. In this registry, all individuals who received any form of benefits are registered by their social security number. We assumed that missing data from the EQ-5D-5L were missing at random and imputed missing values with a multiple imputation model. Missing data was imputed using Multivariate Imputation by Chained Equations (MICE) with Predictive Mean Matching [25]. The imputation model included duration of sick leave at baseline, risk groups from the Keele STarT MSK and ÖMPSQ-SF, work satisfaction, and self-rated health. Ten complete datasets were imputed. Analyses were performed per imputed dataset separately, and the results were then pooled using Rubin’s rules [25]. MICE was performed using SPSS statistics 25 (IBM).

### Cost-effectiveness analysis & cost-utility analysis

In the cost-effectiveness analyses, the outcome measure was sickness absence days, and productivity costs were excluded to prevent double counting. In the cost-utility analyses, productivity costs were included. We used linear regression models, both adjusted and unadjusted for confounders (sex, age, BMI, smoking, education level and physical activity) to analyse disaggregate cost differences. Differences in total costs and effects between treatment groups were obtained from a system of seemingly unrelated regressions that accounted for the potential correlation between costs and effects [26]. These total cost and effect differences were adjusted for baseline and confounders. In both analyses, the incremental cost-effectiveness ratio (ICER) was calculated by dividing the corrected differences in costs by those in effects. To assess uncertainty, we used a bootstrap method with 10,000 replicated datasets. To illustrate the statistical uncertainty surrounding the ICERs, bootstrapped cost and effect pairs were plotted on a cost-effectiveness plane (CE plane) with incremental costs on the y-axis and incremental effects on the x-axis, and on cost-effectiveness acceptability curves (CEACs).

### Cost–benefit analysis

The cost–benefit analysis (CBA) was performed from NAV’s perspective. Costs were defined as intervention costs, and benefits as the difference in total monetized outcome measures between the intervention groups and control group. Positive benefits indicate reduced spending of the intervention groups compared with the control group. Two cost–benefit metrics were calculated: (1) net benefits (NBs), and (2) benefit cost ratio (BCR).

- NB = Benefits – Costs
- BCR = Benefits / Costs

To quantify precision, 95% bootstrapped confidence intervals (CIs) were estimated, using 10,000 replications. Financial returns are positive if NB > 0 and BCR > 1.

### Sensitivity analyses

The following sensitivity analyses were carried out: 1) Complete-case analysis (including participants with complete data only). 2) Uncertainty of the ICER (incremental cost-effectiveness ratio) will be tested by bootstrapping with 5,000 repetitions.

## Results

### Participants

A total of 514 workers participated in the trial, while five participants withdrew, leaving 509 (99%) participants for analyses. No adverse events were reported. A detailed flow chart is shown elsewhere [11]. Table 1 shows that baseline characteristics were similar across the three groups. The median age of participants was 49 years (range 24–66 years) and 57% were women. Totally, 341 participants (66%) worked in full-time positions, and 315 (62%) were on full sick leave at baseline. The mean quality adjusted life years (SD) was 0.58 (0.21). The self-reported level of musculoskeletal health, according to the Musculoskeletal Health Questionnaire (MSK-HQ), was low to moderate with an average score of 27 (on a scale from 0 to 56, where a higher score indicates better health status).

**Table 1:** Baseline characteristics of participants

Characteristic	Missing n (%)	UC (*n* = 174)	UC + MI (*n* = 170)	UC + SVAI (*n* = 170)
Age *(years), median (IQR)*		49 (40–55)	49 (41–56)	49 (41–56)
Women, *n (%)*		94 (54)	99 (58)	100 (59)
Education, *n (%)*				
• Compulsory education		21 (12)	14 (8)	20 12)
• High school		92 (53)	95 (56)	84 49)
• College or university < 4 years		40 (23)	46 (27)	49 29)
• College or university ≥ 4 years		21 (12)	15 (9)	17 (10)
Smokers, *n (%)*		39 (22)	35 (21)	36 (21)
BMI (kg/m^2^), *median (IQR)*	13 (3)	28 (24–31)	27 (24–31)	27 (24–31)
Days of physical activity previous week, *n (%)*	1 (0.2)			
• 0 days		65 (37)	54 (32)	64 (38)
• 1–2 days		46 (26)	43 (25)	39 (23)
• 3–4 days		38 (22)	45 (27)	41 (24)
• 5–7 days		25 (14)	27 (16)	26 (15)
Musculoskeletal health^a^ (0–56), mean (SD)	21 (4)	27 (9)	27 (8)	27 (8)
ÖMPSQ-SF^b^ (≥ 60), *n (%)*		65 (37)	55 (32)	59 (35)
Keele STarT MSK tool (0–12)				
• High risk (≥ 9), *n (%)*		61 (35)	49 (29)	48 (28)
• Medium risk (5–8), *n (%)*		85 (49)	86 (51)	98 (58)
• Low risk (< 5), *n (%)*		28 (16)	35 (21)	24 (14)
Work, *n (%)*				
• Full-time		120 (69)	110 (65)	111 (65)
• Part-time 50–99% of full work hours per week		39 (22)	53 (31)	48 (28)
• Part-time < 50% of full work hours per week		15 (9)	7 (4)	11 (6)
Graded disability pension^c^, yes *n (%)*	5 (1)	15 (9)	12 (7)	9 (5)
Sickness absence days previous year (work days^d^), *median (IQR)*	5 (1)	38 (30–50)	35 (31–50)	36 (26–50)
Mean Quality Adjusted Life Years (SD)^e^	5 (1)	0.57 (0.21)	0.60 (0.21)	0.58 (0.22)
Area of body pain, *n (%)*	14 (3)			
• Lower limb		6 (4)	18 (11)	15 (9)
• Upper limb		30 (18)	30 (18)	30 (18)
• Neck		12 (7)	12 (7)	10 (6)
• Back		34 (20)	42 (25)	43 (26)
• Multisite pain		12 (7)	8 (5)	10 (6)
• Joint disorders		20 (12)	13 (8)	10 (6)
• Fractures		14 (8)	16 (10)	11 (7)
• Other		40 (24)	26 (16)	38 (23)

The distribution was skewed for all continuous variables, except for the MSK-HQ

*UC* usual case management, *MI* motivational interviewing, *SVAI* stratified vocational advice intervention, *N* number of participants, *IQR* inter quartile range, 25^th^ percentile – 75^th^ percentile, *SD* standard deviation

^a^ Measured with the Musculoskeletal Health questionnaire (MSK-HQ)

^b^ ÖMPSQ-SF: The Örebro MSK Pain Screening Questionnaire Short Form (0–100)

^c^ Individuals who work part time and receive a graded disability pension

^d^Lost workdays due to sick leave, adjusted for work hours per week and amount of sick leave

^e^Quality adjusted life years are measured using the EQ-5D-5L

### Cost differences

Mean costs within each study group are presented in Table 2. Total costs were highest in the UC group (€25345 (Standard Error of the Mean (SEM):1226)), followed by the UC + MI ((€22524 (SEM1229)) and the UC + SVAI (€21716 (SEM 1103)). In all three groups, over 90% of costs were due to costs related to absenteeism and productivity losses. The cost of the interventions (MI or SVAI) was less than 0.5% of the total costs.

**Table 2:** Mean cost (EUR) per participant in the various study groups, unadjusted and adjusted mean cost differences between groups

Cost category	UC Mean (SEM)	UC + MI Mean (SEM)	UC + SVAI Mean (SEM)	Comparison 1: UC + MI vs. UCMean (95%CI)		Comparison 2: UC + SVAI vs. UCMean (95%CI)	
				Unadjusted	Adjusted^a^	Unadjusted	**Adjusted**^**a**^
**Intervention costs**	0 (0)	53 (3)	78 (3)	53 (47 to 58)	53 (47 to 58)	78 (72 to 84)	**78 (72 to 85)**
**Total Healthcare costs**	1049 (65)	984 (73)	924 (64)	-65 (-248 to 135)	-66 (-249 to 134)	-125 (-300 to 60)	**-123 (-295 to 72)**
***Prim. healthcare costs***	*919 (60)*	*875 (70)*	*824 (61)*	*-44 (-221 to 143)*	*-41 (-214 to 147)*	*-96 (-259 to 81)*	***-89 (-251 to 97)***
***Sec. healthcare costs***	*129 (13)*	*109 (10)*	*100 (10)*	*-21 (-53 to 9)*	*-24 (-59 to 7)*	*-29 (-62 to 6)*	***-33 (-68 to -2)***
**Absenteeism costs**	22719 (1119)	20413 (1139)	19744 (1039)	-2306 (-5323 to 789)	-2062 (-4924 to 796)	-2975 (-6009 to -85)	**-2194 (-4762 to 501)**
**Productivity losses of unpaid work**	1577 (292)	1075 (297)	971 (212)	-529 (-1289 to – 318)	-522 (-1301 to -316)	-606 (-1340 to 61)	**-619 (-1343 to 27)**
**Total societal costs**	**25345 (1226)**	**22524 (1229)**	**21716 (1103)**	**-2821 (-6084 to 579)**	**-2594 (-5733 to 497)**	**-3628 (-6911 to -488)**	**-2858 (-5701 to 55)**

Total values are depicted in bold font

*UC* usual case management, *MI* motivational interviewing, *SVAI* stratified vocational advice intervention, *SEM* standard error of the mean, *CI* Confidence Interval

^a^Comparisons were adjusted for sex, age, BMI, smoking, education level and physical activity

Intervention costs were higher in the UC + MI and the UC + SVAI groups compared to the UC group (Table 2). All other costs were lower for both intervention groups compared to the UC group. Comparing UC + MI to UC, the UC + MI group had lower total societal costs in the adjusted analysis (-2594 (95% CI -5733 to 497)). Comparing UC + SVAI to UC, total societal costs were in favour of UC + SVAI (-2858 (95%CI -5701 to 55)). Absenteeism was the biggest cost driver.

### Effect differences

Comparing UC + MI to UC, the difference in QALYs was -0.001 (95% CI -0.15 to 0.01) and the reduction in sickness absence days was 5.1 (95% CI -3.3 to 13.5).

Comparing UC + SVAI to UC, the difference in QALYs was -0.002 (95% CI -0.02 to 0.01)) and the reduction in sickness absence days was 7.9 (95% CI -0.04 to 15.9).

### Cost-effectiveness & cost-utility

**Fig. 1:**
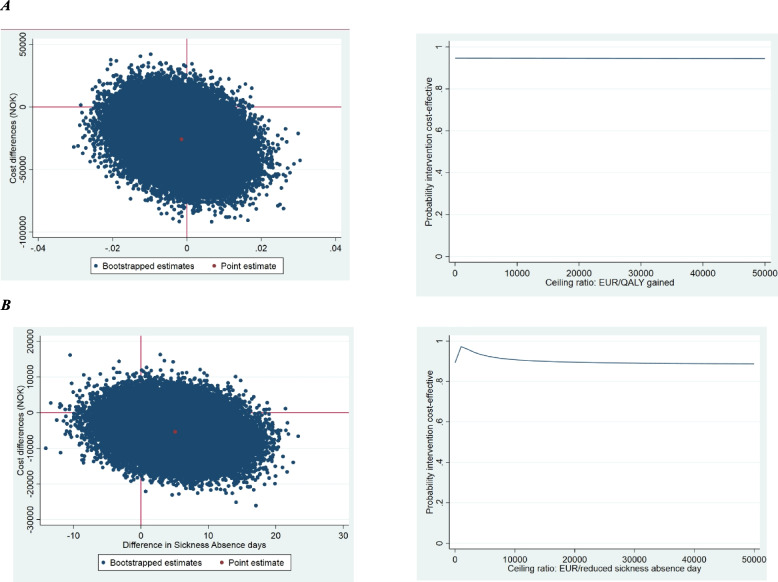
Cost-Utility plane & Cost-Utility acceptability curve for different ceiling ratios (NOK) for qualityadjusted life-years indicating the probability of cost-effectiveness of Motivational Interviewing versus control for workers on (**a**) QALYs or (**b**) sickness absence due to a musculoskeletal disorder

Comparing UC + MI to UC, we found an ICER of 1,756,221 for QALYs, indicating that 1,756,221 EUR would, on average, be saved in the intervention group compared to the control group per QALY gained. Similarly, for the UC + MI group, we found an ICER of 106, indicating a saved average of 106 EUR for each day of sickness absence compared to the UC group. Figure 1 and Table 3 show that most incremental cost-effectiveness (CE) pairs were located on the southern (for QALYs) and southeast (for sickness absence) quadrant(s) of the CE-plane, indicating that the intervention was on average less costly for improving QALYs, and less costly and more effective for reducing sickness absence.

**Table 3:** Cost-effectiveness analysis results (main analysis)

Outcome	Sample size		∆C (95%CI)	∆E (95%CI)	ICER	Distribution CE-plane (%)			
**Comparison 1** **(UC + MI vs. UC)**									
	**UC + MI**	**UC**	**EUR**	**Points**	**NOK/point**	**NE**	**SE**	**SW**	**NW**
**QALYs (0–1)**	169	171	-2580 (-5687 to 612)	-0.001 (-0.02 to 0.01)	1756221	1.1	40.8	53.9	4.2
**Sickness absence days reduction over six months**	169	171	-538 (-1358 to 352)	5.08 (-3.3 to 13.5)	-106	8.5	79.1	10.0	2.1
**Comparison 2** **(UC + SVAI vs. UC)**									
	**UC + SVAI**	**UC**	**EUR**	**Points**	**NOK/point**	**NE**	**SE**	**SW**	**NW**
**QALYs (0–1)**	169	171	-2899 (-5840 to 18)	-0.002 (-0.02 to 0.01)	1553061	0.5	39.9	57.5	2.1
**Sickness absence days reduction over six months**	169	171	-695 (-1459 to -3)	7.9 (-0.04 to 15.9)	-88	2.6	94.8	2.4	0.2

*C* Costs, *E* Effects, *ICER* Incremental Cost-Effectiveness Ratio, *CE-plane* Cost-Effectiveness plane, *NE* Northeast-Quadrant, *SE* Southeast-Quadrant, *NW* Northwest-Quadrant, *SW* Southwest-Quadrant, *CI* Confidence Interval, *UC* usual case management, *MI* motivational interviewing, *SVAI* stratified vocational advice intervention

Comparing UC + SVAI to UC, we found an ICER of 1,553,061, indicating that 1,553,061 EUR would, on average, be saved in the intervention group compared to the control group per 1 QALY gained. Similarly, for the UC + SVAI to UC, we found an ICER of 88, indicating a saved average of that 88 EUR per day reduction in sickness absence compared to the UC group. Figure 1 and Table 3 show that most incremental CE pairs were located on the southeast quadrant of the CE plane, indicating that the intervention was on average less costly and more effective.

**Fig. 2:**
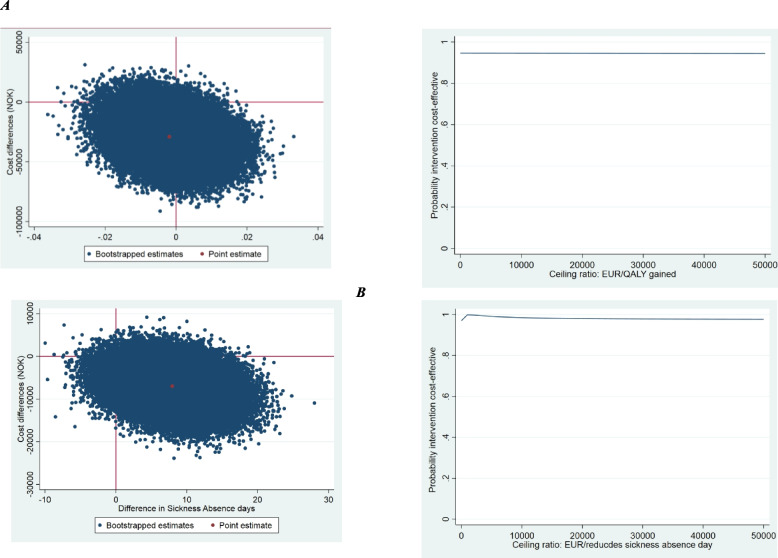
Cost-Utility plane & Cost-Utility acceptability curve for different ceiling ratios (Norwegian Kroner) for quality-adjusted life-years indicating the probability of cost-effectiveness of Stratified Vocational Advice Interventions (SVAI) versus control for workers on (**a**) QALYs or (**b**) sickness absence due to a musculoskeletal disorder

Both CEACs show that the probability of UC + MI and UC + SVAI being cost-effective compared with UC only was higher than 90% for all willingness-to-pay thresholds (Figs. 1 and 2).

### Cost–benefit analysis

The results of CBA were in favour of the MI + UC group (Table 4). The total benefit was 2874 EUR (95% CI -563 to 6299) in the MI + UC group compared with UC group. The mean net benefit (subtracting intervention cost from total benefit) was 2821 EUR (95% CI -617 to 6246) per worker. The BCR (i.e., amount of money returned per Euro invested) was 54 (95% CI-10 to 124). The estimated maximal probability of return was 94.5%, indicating 94.5% probability for NAV to expect a positive return on investment from the intervention.

**Table 4:** Return-on-Investment analysis results (main analysis)

Sample size		Costs (95% CI)	Benefits Total (95% CI)	Net benefit (95% CI)	Benefit Cost Ratio (95%CI)
**Comparison 1** **(UC + MI vs. UC)**					
** MI + UC**	**UC**	**€**	**€**	**€**	**€**
169	171	53 (47 to 58)	2874 (-563 to 6299)	2821 (-617 to 6246)	54 (-10 to 124)
**Comparison 2** **(UC + SVAI vs. UC)**					
** SVAI + UC**	**UC**	**€**	**€**	**€**	**€**
169	171	78 (72–84)	3706 (548 to 7049)	3628 (388 to 6911)	48 (6 to 91)

*C* Costs, *E* Effects, *ICER* Incremental Cost-Effectiveness Ratio, *CE-plane* Cost-Effectiveness plane, *NE* Northeast-Quadrant, *SE* Southeast-Quadrant, *NW* Northwest-Quadrant, *ZW* Southwest-Quadrant, *CI* Confidence Interval, *UC* usual case management, *MI* motivational interviewing, *SVAI* stratified vocational advice intervention, *SEM* standard error of the mean

The results of CBA were in favour of the SVAI + UC group (Table 4). The total benefit was 3706 EUR (95% CI -548 to 7049) in the SVAI + UC group compared with UC group. The mean net benefit (subtracting intervention cost from total benefit) was 3628 (95% CI 388 to 6911) per worker. The BCR (i.e., amount of money returned per Euro invested) was 48 (95% CI 6 to 91). The estimated maximal probability of return as 98.5%, indicating 98.5% probability for NAV to expect a positive return on investment from the intervention.

### Sensitivity analysis

When re-running the analysis on complete cases only, and using 5,000 bootstraps, we observed similar results to those of the main analysis (Appendix 1).

## Discussion

### Main findings

This health economic evaluation assessed adding MI or a SVAI to usual UC for workers on sick leave due to MSDs. In comparison with UC, adding MI had an overall high willingness-to-pay (0.9) and maximum probability of return (94.5%). Financial return estimates were likely to be positive. Adding SVAI to UC showed a high willingness-to-pay (0.9) and maximum probability of return (98.5%). Financial return estimates were highly positive.

### Comparison with other studies

To our knowledge, no previous studies has evaluated the cost-effectiveness of MI for this group of workers on sick leave with MSDs. The effectiveness [11], and cost-effectiveness results showed a similar pattern in which MI on average reduced sickness absence days among people with MSDs [27, 28]. As presented in the effectiveness study [11], a seven-day difference may be considered an important effect. However, the trial was not powered to detect this difference as statistically significant. Large variability in the data may also have reduced the statistical power of the trial, for example participants were heterogenous with respect to diagnosis and previous sick leaves.

The cost-effectiveness results of adding a SVAI to UC supports the findings of a previous trial indicating that vocational advice could lead to reduced absence and cost savings for society [9].

Since 90% of costs in this study were due to sickness absence and productivity losses, this emphasises the importance of RTW interventions. Both MI and SVAI had a high likelihood of being cost-effective and had a positive return on investment. These results are in line with a systematic review on effectiveness and cost-effectiveness studies, showing that for individuals on long-term sick leave due to back pain, interventions including interaction between employees, care personnel and employers appear to be more efficient and cost-effective than other workplace-linked interventions [29].

### Strengths and limitations

This study has several strengths in the implementation of the intervention, as well as analytical strengths. The multi-arm RCT design made it possible to compare two additional interventions with a single UC group, optimising the use of limited research resources. Secondly, detailed national registry data included data for 99% of the trial participants. The analyses were performed based on the pre-registered statistical analysis plan. We used non-parametric bootstrapping methods to determine the imprecision around the estimates, which are recommended to handle skewed cost data. All these attributes support the validity of the findings observed in this study.

The main limitation of this study is the variability of the MI and SVAI intervention, both of which are context- and provider-dependent. Fidelity assessment of the MI intervention showed that the caseworkers had variable proficiency throughout the trial [19]. The process evaluation of the SVAI showed that the intervention was delivered in accordance with the intervention protocol and conversation guide [20]. These findings come from a pragmatic trial, providing results that are valid within the study's specific context. However, these findings need to be replicated in other settings for broader validation. Another limitation of this study was a relatively small sample that could have resulted in non-statistically significant results, because the study was not powered to detect these differences. However, for the main outcomes of the economic evaluation, the cost-effectiveness planes and CEACs, 10,000 bootstraps were used which provide information on the accuracy of the estimate. Thirdly, this study did not include the use of medication in the economic evaluation. However, it is unlikely that this will have had any effect on this study since the interventions were not aimed at medication use, and medication use will most likely only have contributed to a small proportion of the costs [30, 31]. Another limitation was the missing data for QALYs due to non-response and drop-out/lost-to-follow up. To address this issue, we employed multivariate imputation methods, which is recommended practice for dealing with missing values in economic evaluation research [25]. Results of the complete-case analysis, where no imputation was applied, yielded similar findings to the main analysis. Therefore, the degree of dropping out of participants during the follow-up time did not influence the main results.

### Implications for practice and research

Despite higher intervention costs, the two interventions could potentially reduce costs of sick leave if implemented widely. One could also argue that the high intervention costs would decrease over time due to less need for initial training of intervention providers.

Future research could assess the cost-effectiveness of MI and SVAI in other jurisdictions, because the usual care setting as well as costs related to sick leave are highly context dependent. Furthermore, further studies could focus on other diagnoses such as common mental disorders, or workers with MSD and a comorbid serious mental health disorder, which are also frequent reasons for sick leave and interesting groups for stakeholders. In a recent systematic review by Dewa et al. [32] on RTW interventions for mental health related sick leave, they emphasised the importance of conducting more economic evaluations in various disability and health systems.

## Conclusions

Overall, we found tendencies for adding MI to UC to be cost-effective and cost-beneficial compared with UC for workers on sick leave due to MSDs. Similarly, incorporating a SVAI to UC for the same group of workers is also cost-effective and cost-beneficial. Despite the higher intervention costs, implementation of these interventions has the potential to reduce the societal costs related to sick leave.

### Supplementary Information

**Additional file 1:** **Appendix 1.** Cost-effectiveness analysis results (Sensitivity analyses).

## Data Availability

Requests to access data should be addressed to mgrotle@oslomet.no anonymised individual participant data (including data dictionary) will be available on request, to researchers who provide a methodologically sound scientific proposal that has been approved by an ethics committee and by the scientific board of the MI-NAV study.

## Abbreviations

BCR: Benefit cost ratio
CBA: Cost–benefit analysis
CE: Cost-effectiveness
CE plane: Cost-effectiveness plane
CEACs: Cost-effectiveness acceptability curves
CI: Confidence interval
EQ-5D-5L: EuroQol-5 Dimensions-5 Levels
ICER: Incremental cost-effectiveness ratio
ICPC-2: 2^Nd^ edition of the International Classification of Primary Care
IPCQ: Institute for Medical Technology Assessment Productivity Cost Questionnaire
IQR: Inter quartile range
ISPOR: International Society for Pharmacoeconomics and Outcomes Research
MI: Motivational interviewing
MICE: Multivariate Imputation by Chained Equations
MSD: Musculoskeletal disorders
MSK-HQ: Musculoskeletal Health Questionnaire
NAV: The Norwegian Labour and Welfare Administration
NB: Net benefit
NE: Northeast-Quadrant
NW: Northwest-Quadrant
ÖMPSQ-SF: Örebro Musculoskeletal Pain Screening Questionnaire Short Form
QALY: Quality-adjusted life-years
RTW: Return to work
SD: Standard deviation
SE: Southeast-Quadrant
SEM: Standard Error of the Mean
SVAI: Stratified vocational advice intervention
SW: Southwest-Quadrant
UC: Usual case management
