# Supplementary material for: Six-month cost-effectiveness of adding motivational interviewing or a stratified vocational advice intervention to usual case management for workers with musculoskeletal disorders: the MI-NAV economic evaluation
Source: J Occup Med Toxicol. 2023 Nov 14;18:25. doi: 10.1186/s12995-023-00394-2 (PMC10644648; doi:10.1186/s12995-023-00394-2)
Supplement: Supplementary file 1 — Additional file 1: Appendix 1. Cost-effectiveness analysis results (Sensitivity analyses). [file 12995_2023_394_MOESM1_ESM.docx]

***Appendix 1: Cost-effectiveness analysis results (Sensitivity analyses****)*

| COMPLETE CASE ANALYSIS | | | | | | | | | |
| --- | --- | --- | --- | --- | --- | --- | --- | --- | --- |
| Outcome | **Sample size**  **Outcome** | | **∆C (95%CI)** | **∆E (95%CI)** | **ICER** | Distribution CE-plane (%) | | | |
| Comparison 1  (Int. group 1 vs. Control) | | | | | | | | | |
|  | **UC+MI** | **UC** | **EUR** | **Points** | **EUR/point** | **NE** | **SE** | **SW** | NW |
| QALYs (0-1) | 92 | 114 | -2378 (-6757 to 1999) | -0.002 (-0.02 to 0.01) | 1,455,866 | 3.9 | 38.1 | 48.2 | 9.8 |
| \| **Comparison 2**  **(Int. group 2 vs. Control)** \| \| \| \| \| \| \| \| \| \| \| \| --- \| --- \| --- \| --- \| --- \| --- \| --- \| --- \| --- \| --- \| --- \| \|  \| **UC+SVAI** \| **UC** \| **EUR** \| **Points** \| **EUR/point** \| **NE** \| **SE** \| **SW** \| **NW** \| | | | | | | | | | |
| QALYs (0-1) | 99 | 114 | -3364 (-7298 to 607) | -0.003 (-0.02 to 0.01) | 10,746,135 | 1.0 | 35.4 | 59.9 | 3.7 |

| 5000 bootstraps | | | | | | | | | |
| --- | --- | --- | --- | --- | --- | --- | --- | --- | --- |
| Outcome | **Sample size**  **Outcome** | | **∆C (95%CI)** | **∆E (95%CI)** | **ICER** | Distribution CE-plane (%) | | | |
| Comparison 1  (Int. group 1 vs. Control) | | | | | | | | | |
|  | **UC+MI** | **UC** | **EUR** | **Points** | **EUR /point** | **NE** | **SE** | **SW** | NW |
| QALYs (0-1) | 169 | 171 | -2579 (-5687 to 569) | -0.01 (-0.02 to 0.01) | 1,756,220 | 1.2 | 40.5 | 54.3 | 4.0 |
| Sickness absence days over six months | 169 | 171 | -537 (-1371 to 345) | 5.08 (-3.3 to 13.5) | -106 | 8.6 | 79.4 | 9.8 | 2.1 |
| \| **Comparison 2**  **(Int. group 2 vs. Control)** \| \| \| \| \| \| \| \| \| \| \| \| --- \| --- \| --- \| --- \| --- \| --- \| --- \| --- \| --- \| --- \| --- \| \|  \| **UC+SVAI** \| **UC** \| **EUR** \| **Points** \| **EUR /point** \| **NE** \| **SE** \| **SW** \| **NW** \| | | | | | | | | | |
| QALYs (0-1) | 169 | 171 | -289 (-5833 to 32) | -0.002 (-0.02 to 0.01) | 1,553,061 | 0.5 | 39.6 | 57.8 | 2.1 |
| Sickness absence days over six months | 169 | 171 | -695 (-1464 to -6) | 7.9 (-0.07 to 15.9) | -77 | 2.6 | 94.7 | 2.5 | 0.2 |
